# The Challenges of Decolonising Sustainability and the Environment in Development Studies (DS)

**DOI:** 10.1057/s41287-025-00700-0

**Published:** 2025-04-22

**Authors:** Lyla Mehta

**Affiliations:** 1 Institute of Development Studies at the University of Sussex, Brighton, UK; 2 Norwegian University of Life Sciences, Aas, Norway

**Keywords:** Development studies, Sustainability, Environment and development, Decolonising sustainability and the environment, Decolonisation, Decoloniality

## Abstract

This think-piece makes a case for addressing the colonial roots of sustainability. It examines how enduring colonial mechanisms and biases have led to certain forms of value and valuing, problematic views of ‘pristine nature’ and processes of extractivism. These in turn have led to dispossession and violence, especially for Indigenous and marginalised communities in the majority world. It explores how studies on the environment and sustainability have sought to implicitly or explicitly challenge these colonial biases and their impacts. Researchers working on the environment, gender and sustainability have brought together Development Studies (DS) with science technology studies (STS), (feminist) political ecology, anthropology and feminist epistemology. This has resulted in strong engagements with the politics of knowledge, the colonial roots of environmental problems and the need to lift the perspectives and voices of historically marginalised groups to promote alternative ways of doing and understanding development and nature/society relations. Researchers working in other fields of DS could do more to draw on these diverse perspectives, especially since epistemic and material inequalities and power structures are interlinked and mutually reinforcing. I also focus on the dangers of decolonisation becoming a buzzword without much change in actual practices in ways of working and collaborating.

## Résumé

Cet article de réflexion plaide pour une prise en compte des racines coloniales de la durabilité. Il examine comment les mécanismes et préjugés coloniaux persistants ont conduit à certaines formes de valeur et d'évaluation, des visions problématiques de la 'nature vierge’ et des processus d'extractivisme. Ceux-ci ont à leur tour conduit à la dépossession et à la violence, en particulier pour les communautés autochtones et marginalisées dans le monde majoritaire. Il explore comment les études sur l'environnement et la durabilité ont cherché à contester implicitement ou explicitement ces préjugés coloniaux et leurs impacts. Les chercheurs travaillant sur l'environnement, le genre et la durabilité ont réuni les études de développement (ED) avec les études de la science et de la technologie (EST), l'écologie politique (féministe), l'anthropologie et l'épistémologie féministe. Cela a abouti à des engagements forts avec la politique de la connaissance, les racines coloniales des problèmes environnementaux et la nécessité de mettre en avant les perspectives et les voix des groupes historiquement marginalisés pour promouvoir des alternatives de faire et de comprendre le développement et les relations nature/société. Les chercheurs travaillant dans d'autres domaines des ED pourraient faire plus pour s'appuyer sur ces perspectives diverses, en particulier puisque les inégalités épistémiques et matérielles et les structures de pouvoir sont interconnectées et se renforcent mutuellement. Je me concentre également sur les dangers de la décolonisation devenant un mot à la mode sans beaucoup de changement dans les pratiques réelles de travail et de collaboration.

## Introduction

Calls to decolonise development have been around for decades, mostly due to anti-imperialist movements across the majority world stressing emancipation and freedom. However, these calls have grown in the twenty-first century, not least due to the growing awareness of how steeped development and development studies (DS) are in coloniality and colonial process (cf. Quijano 2007; Narayanaswamy 2024). In the past decade, different social movements from #BlackLivesMatter, alternative calls from global South movements (e.g. Ubuntu and Buen Vivir) as well as coalitions around pluriversality (cf Kothari et al. 2019) have highlighted the asymmetrical political, socio-economic, racialised and cultural processes rooted in colonial imperial histories. These have led to structural and power imbalances as well as epistemic and material forms of injustice, vulnerabilities and violence for countries in the majority world as well as brown and black people across the globe.

This think-piece makes a case for addressing the colonial roots of sustainability. It focuses on how colonial mechanisms and biases have led to certain forms of value and valuing, problematic views of ‘pristine’nature’ and processes of extractivism which have led to dispossession and violence, especially for Indigenous and marginalised communities and people of colour. The piece explores how studies on the environment and sustainability in DS have sought to implicitly or explicitly challenge these biases and their impacts. This is a think-piece, not a review paper and due to space constraints I can only provide a selective overview of the key trends and studies. Despite this limitation, I hope to show that critical conceptual and empirical work on nature/society relations in development contexts has played a significant, yet overlooked, role in changing DS. Researchers working on the environment, gender and sustainability have deployed diverse frameworks bringing together DS with fields such as science and technology studies (STS), (feminist) political ecology, anthropology and feminist epistemology. This has resulted in a strong engagement with the politics of knowledge, the colonial roots of environmental problems and the need to lift the perspectives and voices of historically marginalised groups to promote alternative ways of doing and understanding development and nature/society relations. Researchers working in other fields of DS could do more to draw on these perspectives in their work, especially since material and epistemic power structures are interlinked and mutually reinforcing. Yet, whilst critiques of coloniality have recently become more explicit, there are also dangers of decolonisation becoming a buzzword without much change in actual practices in our ways of working and collaborating. The article is structured as follows: I start by outlining four key colonial biases around sustainability and the environment and then provide a selective journey of how some works DS are seeking to decolonise and challenge these practices. The final section discusses how we can and should do better in decolonising our practices and ways of working.

## The Colonial Roots of Studies on Sustainability

Since the Brundtland Commission advanced the concept of sustainable development in 1987, a lively strand in DS has engaged with the linkages between environment, sustainability and development. This remained fairly niche until the 2030 Agenda, with its 17 Sustainable Development Goals (SDGs) goals, mainstreamed sustainable development and also questioned conventional boundaries between the global South and global North due to the emphasis on universality (Aghajanian and Allouche 2016). In everyday life, sustainability refers to the ability to be maintained at a certain rate or level and usually the balance between the use (or depletion) of natural resources and (capitalist-based) growth. The term ‘sustainability’ was first coined in an environmental context in the eighteenth century by a German forester, Hans Carl von Carlowitz, to prescribe how forests should be managed on a long-term basis (see Leach et al., 2015; Mehta 2022). Whilst engagements with sustainability may seem as if they are neutral ways to study and describe the relationships between humans and nature, in reality, they are deeply shaped by the legacies of colonisation and coloniality. These have shaped patterns of thinking, institutional structures, and exploitative flows of money and resources from the times of Europe’s colonies and empires. These patterns have given rise to four mechanisms of colonial biases and practices that persist even today. They are as follows:

First, the colonial early emphasis on conserving economically valuable natural resources led to the focus on certain kinds of *value and valuing,* namely the monetary and use-value over cultural and Indigenous values. This started during imperial and colonial expansion and was consolidated during colonial and post-colonial periods. Thus, local people and their practices were labelled as primitive or environmental destroyers, justifying their removal, restriction or re-education in the name of more ‘efficient’ (read: profitable) resource use (see Leach and Means, 1996).

Second, as European colonisation expanded, many practices, policies and interventions in forestry, agriculture and mining in the so-called ‘tropics’ were geared to *extractivism and profits* for European colonial states (Guha and Gadgil 1989). This was a time of rapid exploitation of natural resources in most parts of Africa, Asia and Latin America. The social consequences of these policies and practices were often devastating, accompanied by slavery, racialised discrimination and the grabbing of land and livelihoods from Indigenous people and local communities, and exploitative and degrading labour practices (Hernandez 2022).

Third, alongside this widespread destruction and exploitation, colonial environmentalism included another, parallel desire: *the aesthetic and moral urge to preserve an imagined pristine nature and wilderness in the ‘tropics’*. This continues in modern-day ‘fortress conservation’ (see Brockington 2002), where large tracts of land are fenced off against perceived threats from local people and still embedded in mainstream notions about African nature. To illustrate, we can review the ideas about wilderness in some of the documentaries of David Attenborough (Oxley 2020) or Prince William claiming that human population was a huge problem for African wildlife.^1^

Fourth, as feminist scholars have shown through the notion of the Plantationocene (cf Haraway 1988), the colonial era was marked by *dispossession and violence*. The promotion of certain types of agriculture and forestry, including scientifically managed plantations of certain crops (e.g. timber and rubber), led to unsustainable monocultures of exotic crops that undermined biodiversity, and supported slavery, indentured labour, and patriarchal supremacy (Tsing 2012). They also involved dispossession and atrocities against peoples of colour. In all the mechanisms of coloniality described above, material and epistemic power structures have reinforced each other, highlighting their interlinkages. For example, ideas of an ‘imagined pristine nature’ led to and justified material vulnerabilities such as dispossession and the loss of livelihoods for local forest-dwellers and Indigenous people. Similarly, material inequalities between colonial powers and colonised nations justified the epistemic and other forms of violence that legitimised slavery, land grabs, biodiversity loss via monocultures and white supremacy.

Borders and leaders have changed, but forms of so-called ‘sustainable development’ that dispossess people of their rights and livelihoods still abound. Colonial biases and legacies are alive and strongly persisting in the here and now. And they are found in the offices of aid donors, academic courses and bureaucracies in both the majority and minority worlds. These are used to justify social and environmental control, securitisation, and the perpetuation of unequal social, racialised and power relations. The present-day justifications are different, however. Today in the name of tiger and wildlife conservation, about 400,00 people from 90,000 *Adivasi* (tribal) families may well be threatened with evictions from forest tiger reserves across India based on exclusionary colonial forest practices (Bijoy 2024). Modern projects are carried out in the name of green energy and climate mitigation—including solar parks and wind farms in India—with some of them perpetuating negative environmental, social and gendered injustices (Kaur 2022). All these issues raise challenging questions for researchers working on sustainability and the environment in DS, an area of work which many hope should support well-being and justice around the world. I now turn to some attempts in DS to address these problematic colonial mechanisms and biases and their implications for current policy and practice.

## How has DS Addressed the Colonial Biases Around Sustainability?

When DS emerged as countries in the majority world were coming out of colonisation, it strongly emphasised economic development and orthodox development economics (see Sumner 2022; Nugent and Yotopoulos 1979). Mainstream development thinking was dominated by the modernisation paradigm ‘with emphasis on economic growth, industrialization, structural differentiation and functional specialization’ (Knutsson 2009, p. 11), and notions of successful growth and modernisation ‘trickling down’ to backward areas (McKay 2008). Big top-down infrastructure projects such as large dams (famously called ‘temples’ of modern India by Nehru), were the symbols of this era. In the name of ‘development’, the massive environmental, gendered and social impacts of these projects were mostly ignored and seen to be a price to pay in return for cheap power generation and irrigation (see Mehta 2005). Today DS is a heterogenous and vibrant field that embraces multi- and interdisciplinary perspectives and normative leanings concerned with social, gender and environmental justice, power relations and addressing poverty and inequality. There is increasing awareness around the complexities of the colonial roots of the discipline and the historical and colonial legacies of development studies institutions (see Kothari 2019). This is because many DS institutions across Europe were established as colonial and post-colonial institutions to support their governments’ assistance to the former colonies (see Aghajanian and Allouche 2016). In the UK and across Europe, DS institutions have today moved from being dominated by white male largely Oxbridge-educated economists to being far more heterogenous, diverse and sensitive to the politics of knowledge, power, gender and representation and more attuned to their own history of coloniality. Significant research on gender and environment and development since the 1980s and 1990s has played a key role in this shift.^2^ Yet, of course, inconsistencies remain, especially in terms of funding and governance structures (see below) as well as silence on issues concerning race and racism (Patel 2020). 

I believe that critical conceptual and empirical work on nature/society relations in development contexts has played a significant but overlooked role in changing DS. The strength of this engagement has been to focus not just on the historical legacies of colonialism but more importantly to highlight links between material and epistemic injustices. For example, the fear of the ‘wasteful’ and ‘irrational’ practices of African land users led to environmental degradation narratives in colonial Africa amongst policy makers and scientists that persist to this day in governments and aid agencies (see Leach and Means 1996). In the water domain, studies looking at dam-building in the context of drought and flood management measures in India have highlighted how colonial and post-colonial water and irrigation management strategies have ignored the natural rhythms of both deltas and drylands. Instead, they have largely focussed on making these landscapes ‘natural resources’ for capitalist accumulation rather than building on and strengthening local livelihood strategies and knowledges. These trends continue today, having devasting social and ecological impacts (D’Souza 2006; Mehta 2005). Researchers working on deforestation from West Africa to South Asia have argued that much of the current problems can be linked to unchanged colonial forest policies that have sought to nationalise ancestral forest lands of local forest-dwellers rather than recognise the inherent rights of forest communities to their forests and lands (Enuoh and Bisong 2015; Fairhead and Leach 1996; Gadgil and Guha 1989).

Challenging these dominant narratives, practices and programmes is only possible through inductive and longue durée research together with local people and local researchers drawing on a diversity of frameworks and approaches. These diverse approaches combine the DS focus on poverty and livelihoods with: environmental history to understand the colonial roots of current environmental problems (Damodaran et al. 2021); political ecology’s concern to marry the power of discourse and its linkages with issues of access, control and rights to resources (Peet and Watts 2004); feminist political ecology’s focus with the gendered nature of resource use with links to intersectionality, subjectivities and affective relations with the environment (Nightingale 2011); STS’ concern with co-production as well as the role of politics and power in environmental discourses (Jasanoff 2004); and post-colonial and feminist decolonial perspectives that call for a greater attention to positionality and praxis (Schöenberg 2019). Overall, the emphasis is on creating policies and practices that encourage an ethics that is ‘not structurally implicated with the consumption of life of earth and others’ (Icaza 2022, p. 52).

Studies focussed on environmental aspects of development have thus sought to both implicitly and explicitly challenge the colonial mechanisms, legacies and biases outlined in the previous section concerning value, denigration of local users and their practices and the associated processes of extraction and dispossession. These imbalances and their associated practices are increasingly being decolonised in several ways: First, this means engaging in scientific enquiries and methodologies that lift hidden invisible and Indigenous perspectives, ways of living and being, and alternative visions around value and sustainability that are missing in the dominant scientific framings shaped by Western world views (Smith 2021). This is because official state programmes often dismiss local environmental practices (from forestry to water, soil management and pastoralism) that can promote biodiversity, environmental regeneration and local livelihoods (Mehta 2005; Rocheleau 2011) and are often best suited to deal with complexity, uncertainty and climate extremes (cf. Scoones 2023). Through these multiple approaches and perspectives, the aim is to strive for cognitive and epistemic justice, and allowing for the perspectives and experiences of Indigenous and marginalised peoples and their views of nature and resources to be the starting point of scientific analyses.

Second, it means pushing back against destructive and exploitative environmental practices that continue to colonise nature and people, and questioning the logic of colonial modernity that reproduces power and privilege in environment and development processes based on notions of superiority, domination and extraction. As Arora and Stirling (2023) powerfully argue, challenging the logic of colonial modernity can enable convivial pluriversality and allow different worlds to flourish together.

Third, it is important to actively counter the colonial legacies that, to this day, perpetuate the notion that certain lives and livelihoods are ‘unproductive’, ‘destructive’ and other similar descriptions. An example of this is grasslands, often considered to be ‘wastelands’ in South Asia. Colonial rulers treated such lands as inferior and sought to make them ‘productive’ via monoculture plantations and irrigated agriculture. This undermined biodiversity and local resource users such as pastoralist communities, who have sustainably used this landscape for their livelihoods, whilst also contributing to regional biodiversity. Post-colonial India continues to view drylands and grasslands as ‘wastelands’ and barren spaces that need to be diverted for ‘development’ and commercial purposes. Furthermore, local users of these resources, such as pastoralists, are vilified (Srivastava and Mehta 2021). As argued by Paul and Vanak (2020), Scoones (2023) and others, restoring drylands and grasslands is an effective way to combat climate change as these landscapes store large amounts of carbon below ground and enhance biodiversity.

This means seeking out methodologies and approaches that reverse processes that marginalise non-western and Indigenous knowledge systems (see Chilisa 2017). For example, in the TAPESTRY^3^ project, a team of natural scientists, social scientists and practitioners worked with herders through participatory mapping and the use of satellite imagery, tracking changes over time. In doing so, the aim was to validate the herders’ knowledge that camels are not destructive to coastal and mangrove ecologies but instead live synergistically with mangroves. The evidence thus contradicted the forest department and state scientists’ views that camels were destructive for their local habitats (see Srivastava and Mehta, 2021). Rather it suggested that camel grazing can ultimately help the biodiversity of the drylands and mangrove regeneration (Ohte et al. 2025).

Such co-produced processes can enable collaborative knowledge production in a diversity of contexts. Ideally co-production should address wider societal and power dynamics and go beyond just bringing different groups of people together to create new knowledge. Rather, knowledge is co-produced alongside the social orders in which it is shaped and driven (cf. Jasanoff 2004), which can help ensure that the voices of disenfranchised people make it to formal research papers and policy processes. It can also help gain ‘Two-eyed seeing which is learning to see from one eye with the strengths of Indigenous ways of knowing and from the other eye with the strengths of Western ways of knowing and to using both of these eyes together’ (Bartlett et al. 2012, p. 335) as a way to bring together western methods and approaches with Indigenous and local knowledges (see also Peltier 2018).

Fourth, this also means challenging tools and methods that shape dominant and mainstream nature-society narratives and practices. This, along with co-producing knowledge, does not only help reframe these landscapes; they also help us to question the indices, tools and scientific instruments used to understand nature–society relations and understand the ecological impacts of human activity. As argued by Requena-i-Mora and Brockington (2021), these scientific instruments are not neutral; rather, they reflect the worldviews of their creators that can also colonise minds, landscapes and people and promote green colonialism. Thus, indices such as the environmental performance index, or ‘ecological footprints’,^4^ can serve to reproduce the unequal power relations and practices between rich and poor, and powerful and powerless people. These can help the rich and powerful to look ‘environmentally clean’, hiding injustices and ecological distributional conflicts, and enabling colonial forms of environmental governance.

## The Challenges that Remain

I have demonstrated so far that engagements with sustainability and the environment in DS have both implicitly and explicitly challenged coloniality and its pernicious impacts and in doing so have explored news ways of conducting research and challenging both epistemic and material inequalities and power structures. Given that climate and environmental extremes are affecting all aspects of development, the impacts of environmental and climate change are now more mainstream (cf. Scholz 2019). Increasingly, they are being addressed by most scholars in DS (including those who would not normally address environmental issues). However, this often takes place in superficial and reductionist ways without looking at the complexities around causality, attribution, tenure, access, existing vulnerabilities and how climate change cannot be isolated from wider socio-historical, economic and political legacies and trajectories of change (Mehta et al. 2022). Thus, the wider DS community should perhaps also engage with the long tradition of scholarship on environment and development while engaging with contemporary climate change challenges.

It is also worth noting some of the pitfalls and dangers of decolonial approaches. Currently, decolonisation is a buzzword. I recently reviewed about 15 proposals for a prestigious UK funding call and almost all the proposals spoke about decolonising research whilst retaining European and western frameworks and not really addressing how they would decolonise ways of working. Decolonisation has also been co-opted by some far-right actors. For example, Hindu nationalists are claiming to decolonise India by elevating certain ways of thinking in health and education systems and rejecting the use of English in official speeches. But in doing so they are not only making absurd and dangerous claims (e.g. that cow’s urine can be a cure for Covid-19^5^) but also erasing Islamic and Mughal history from textbooks, elevating Hindu figures and resorting to culture wars that target minorities and vulnerable groups (Dhingra 2023).

Whilst striving for equitable partnerships between majority and minority world researchers and co-producing research together with local people and NGOs is very important, in practice power asymmetries, massive differences in institutional capacities and dangers of co-option remain. Critical social science engagement is being discouraged in many parts of Africa, not least because the World Bank de-funded higher education, tallying with the interests of some governments (see Mkandawire 2011). This trend is also increasingly observed in Europe and the USA. Despite many good intentions, knowledge asymmetries and divide exist (see Melber 2019), especially when it comes to Africa. As acknowledged by the IPCC (2022), multiple studies on climate change in Africa, a continent that is hugely vulnerable to climate extremes, are being conducted without full African participation which will invariably impact applicability on the ground. Finally, mainstream adaptation and mitigation responses to climate change in both the global North and South often resort to using the market mechanisms to address climate change and sustainability challenges and deny the colonial roots of the climate crisis (Sultana 2022). This kind of greenwashing demonstrates the persistence of hegemonic knowledges and practices, rather than embracing other ways of living, knowing and being.

Overall, we could do much better in decolonising our practices, institutional arrangements and projects, as well as our ways of working. This means taking seriously multiple dimensions of power and privilege, race, gender, sexuality, nationality, neurodivergence, passport and visa privilege, as well as institutional and socio-economic differences in research groups (Trisos et al. 2021). This calls for deep listening, being humble, reflexive, and fighting institutional racism and biases. It could mean questioning the invisibility of whiteness and the white gaze, which can make institutions in the global North immune to colonial legacies in environment and development, as well as continual forms of subtle (and not so subtle) discrimination practices against non-Western colleagues (Pailey 2020). As highlighted by Gani and Khan (2024), reflexivity merely ‘performed’ in positionality statements can also be a form of coloniality. But reflexivity in relation to practice means scrutinising and being self-aware and critical of one’s own epistemology and role in the perpetuation of power relations in the research process. It also entails acknowledging our own multiple positionalities, vulnerabilities and emotions as we navigate power hierarchies whilst working with marginalised people in charged research contexts (see Armijos et al. 2024). It is equally important for non-white researchers like me to subject ourselves to this gaze as well. It means seeking to understand the invisibility of different forms of privilege that we grew up with. In my case, this means addressing head on my own status as a Parsi Indian from Bombay (now Mumbai) and also the different class/ caste/ ethnic biases I may have internalised through socialisation, cultural and educational capital and my role in perpetuating these. Many Indian-born/origin DS scholars in Europe also need to look carefully at their mostly *savarna* (‘higher’/oppressor) caste status and what this means for knowledge production and research practice.

We thus need to decolonise our research projects and partnerships. We must ask whether we are perpetuating unequal terms of doing research—since people and institutions in the global North largely control the flow of money and resources, and can still perpertuate colonial mindsets and biases in the way projects and questions are framed (see Aboderin et al. 2023). The low salary remuneration and the lack of research budgets for global South intellectuals and academics, mean that many of them resort to consultancies. Populist governments in Europe are actively moving away from their hitherto generous aid commitments (e.g. Sweden, and the Netherlands). This was even before the recent collapse of USAID under Trump and further cuts to the UK aid budget.  All these issues limit the ability of academics to commit time and support to alternative knowledges and practices. Whilst not common in the UK, long-term institutional collaborations and funding arrangements (as found in Scandinavia) between European and global South institutions can encourage meaningful student and staff exchanges, long-term partnerships and mutual learning. Increasingly, funders are calling for equitable funding partnership models or also requiring leadership by southern institutions. These are steps in the right direction, moving away from hitherto widespread research and teaching models based on patronising, colonialist and unidirectional notions of ‘capacity development.’

Finally, the DS community cannot claim to be working on the linkages between global poverty, inequalities, conflict and environmental destruction and yet keep silent when war is causing human-induced famine, scholasticide, ecocide and even genocide in Gaza. Other less publicised but equally tragic genocides that have had devastating socio-environmental consequences have also taken place in Tigray, Sudan, Myanmar and the Congo. In this context, solidarity statements and actions from the Development Studies Association (DSA, UK), the European Association of Development Research and Training Institutes (EADI) and other European development associations are long overdue and would contribute to calls for justice. After all, European donors and countries are complicit in aidwashing and, simultaneously supporting the military-industrial complex that perpetuate these wars (Bhriain and Akkerman 2024).^6^ Scholars working on linkages between development, the environment, racism and (settler) colonialism therefore need to call out and make every possible effort to resist the structures that perpetuate these grave injustices, as well, as the mass destruction of life itself.

## Data Availability

Not applicable.

## Footnotes

1 See efforts by anthropologists such as John Aini and Paige West to decolonise conservation. Decolonizing Conservation—Paige West (paige-west.com).

2 See Harcourt (2019) for how the gender lens has changed DS. Leach and Scoones (2025) provide a 30-year overview of key articles from the IDS Bulletin on environment and development, also describing the key contributions and contours of this trajectory.

3 https://tapestry-project.org/

4 https://epi.yale.edu/

5 https://www.dw.com/en/hindu-group-hosts-cow-urine-drinking-party-to-ward-off-coronavirus/a-52773262

6 The role of growing powers in the Middle East in sustaining many wars should also be acknowledged (see Ariel and Alaaldin 2022).
